# Subjective and Electroretinographic Dynamics of Light Adaptation in the Human Visual System

**DOI:** 10.3390/vision2010010

**Published:** 2018-02-19

**Authors:** Friederike M. Thoss, Simone Ballosek, Bengt Bartsch, Franz T. Thoss

**Affiliations:** Carl-Ludwig-Institute of Physiology of the University of Leipzig, Liebigstr. 27, 04103 Leipzig, Germany

**Keywords:** rapid light adaptation, discrimination threshold, increment threshold, direct current electroretinogram, glare

## Abstract

The excitation of the visual system increases with increasing retinal illumination. At the same time, the sensitivity of the system decreases (light adaptation). Higher excitation automatically results in a lower sensitivity. This study investigates whether this antagonistic relationship between excitation and sensitivity also applies to the dynamic case, that is, during the transition to a higher excitation level after a sudden increase in retinal illuminance. For this purpose, the courses of the subjective and the electroretinographic threshold in the transitional period during and after a step of the adaptation illuminance were investigated by means of a special light-stimulation system. The investigation was carried out on 9 (subjective threshold) and 12 (electroretinographic threshold) subjects. As a measure of the course of the excitation during this time, the response ERG on the adaptation step was recorded. With the step in adaptation light, the thresholds show a rapid increase, which starts already about 0.1 s before the step. This is followed, within the next second, by a threshold decrease to a new plateau above the initial level. The comparison between the response ERG on the adaptation step and the course of the electroretinographic increment threshold during this time shows a broad agreement between the two courses. Thus, it can be assumed that the sensitivity of the visual system also follows the excitation in the dynamic case. In addition, the investigation shows that the glare experienced after a step in illuminance apparently shows great subjective differences.

## 1. Introduction

In a series of studies, the change in the discrimination threshold was measured after a sudden increase in illuminance [1,2,3,4,5,6,7,8,9]. The authors found consistently that after a very rapid increase of the threshold, a slower decrease followed to a new level above the initial level. The rapid rise of the threshold is, according to [5], caused by “masking”, i.e., the masking of the test stimulus by the illumination intensity step.

In the case of complete adaptation, the discrimination threshold depends on the actual retinal illuminance (Weber’s law) and thus on the strength of the excitation. This finds its expression, e.g., in the Steven’s power function for the relationship between the brightness sensation E and the illuminance I: E = k·I^0.33^. The steepness of this function corresponds to the sensitivity at the respective illuminance, and thus to the reciprocal of the discrimination threshold; the higher the excitation, the lower the slope and the higher the threshold. Our hypothesis is as follows: If this also applies to the dynamic case, i.e., during and after a step of the illuminance, the cause for the “masking” is elucidated; the threshold follows the overshoot of the excitation, which always occurs after a step of illuminance. This should be true both in terms of subjective sensitivity or electroretinogram (objective) measures.

In order to test our assumption, we repeated the measurement of the subjective threshold with a collective of test subjects and subsequently investigated the course of the ERG increment threshold with a second collective in connection with a step in illuminance. In addition, the electroretinographic response to the illuminance step was recorded as a measure of the excitation. The comparison of the two ERG-related courses should show whether or not our assumption is correct.

All the investigations were performed monocularly at the left eye. The applied light stimulation system produced a constant adaptation light of 30°, 0.5 lx; a 10°, 2.5 lx adaptation light step of 5 s duration, which repeated continuously for 10 s; and a 5° test stimulus of 0.1 s duration, which could be shifted in time relative to the adaptation pulse (for details see Materials and Methods).

## 2. Materials and Methods

### 2.1. Light Stimulation Equipment

**Figure 1:**
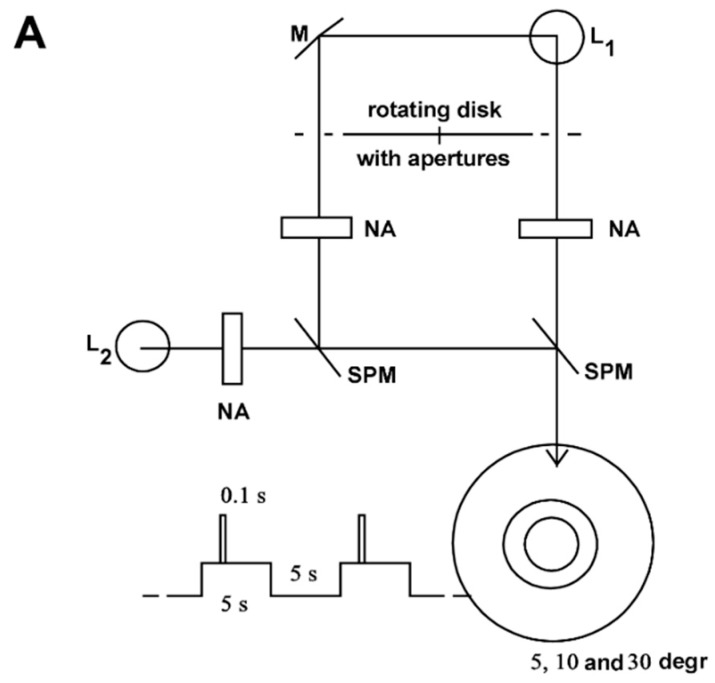

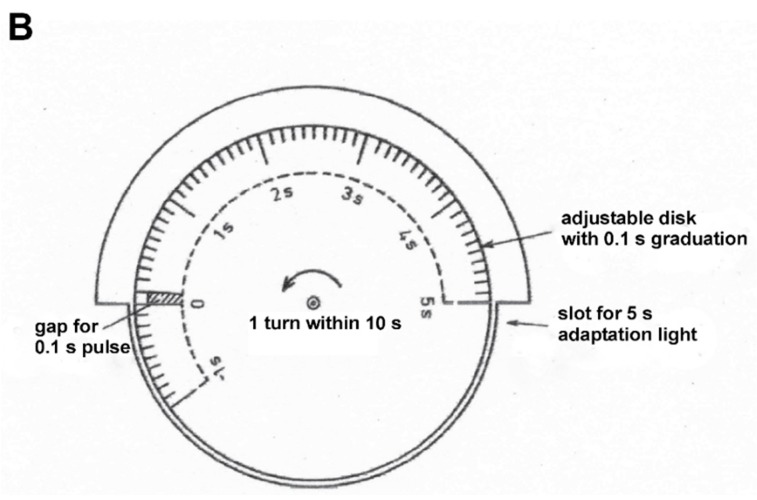
Equipment for generating adaptation and stimulation light. (**A**) The light source L_1_ generates the adaptation pulse via the mirror M and the test stimulus directly. The respective strengths are set by neutral filters at NA, the field sizes of 10° and 5° by diaphragms. The light source L_2_ generates the continuous adaptation light. Its intensity is adjusted by a neutral filter at NA and its field size (30°) by a diaphragm. The beam paths are combined at the semi-transparent mirrors SPM. L_1_ and L_2_ are 12 V, 50 W incandescent lamps; (**B**) Disc combination for the generation of adaptation pulse and stimulus light. The disc makes one revolution in 10 s, thereby clearing the beam path for the adaptation pulse for 5 s, and that for the stimulus light for 0.1 s. The disc consists of 2 single discs. Their twisting against each other makes it possible to change the time interval between adaptation step and stimulus light from −1.0 s (stimulus light 1 s before adaptation step) up to +5.0 s (stimulus light 5 s after adaptation step).

Figure 1 shows the equipment for generating adaptation and stimulus light. A constant adaptation light of 0.5 lx, 30°, an adaptation light step of 2.5 lx, 10°, and a test stimulus of 5° fell on the retina of the test person. (For the transparency of the eye media, a value of 0.8 was assumed for the wavelength range in question.) The time courses of the adaptation pulse (5 s) and test stimulus (0.1 s) were generated by the rotating disk shown in Figure 1B. This type of disk combination had already been used by Crawford [1].

The examination room was lit only by a weak red light. The subject was sitting in front of the equipment, supported by a headrest with the left eye on the eyepiece. When passing through the pupil, the bundle of light was so narrow that the pupil reaction had no influence on the illuminance on the retina (Maxwellian view, for example [10]). The test subject fixed the crosshair in the eyepiece throughout the entire examination. Thus, it was ensured that the test stimuli were mapped on the Macula lutea with the centre in the Fovea centralis.

### 2.2. Measurement of the Subjective (Discrimination) Threshold

#### 2.2.1. Subjects

The selection of subjects was performed arbitrarily, the only prerequisite for participation in the tests was a normal visual system. Myopia and/or hyperopia were corrected by the ocular of the device. A total of nine subjects (seven females, two males) participated in the experiments. The age ranged between 21 and 49 years.

#### 2.2.2. Procedure of the Tests

Adaptation to the test conditions took place for 15 min before the threshold measurement was started. The brightness of the test stimulus was initially selected in such a way that it was clearly recognized by the subject. The stepwise reduction was then performed by means of neutral filters, always to about 70% of the previous test strength (staircase method). Each test stimulus was presented to the subjects 13 times. The first three presentations were used to adapt to the test situation. Then the test subject replied to every stimulus that she/he perceived with “seen” or “yes”. The threshold was achieved when five yes replies were made [11].

The threshold was identified at the following times before and after the step of the adaptation light: −0.5; −0.2; −0.1; 0.0; +0.1; +0.2; +0.3; +0.5; +1.0; +1.5 s.

The times indicate the time intervals between the test stimulus and the adaptation light step. 0.0 s means synchronicity of the two events. A negative sign means that the test stimulus appeared by the specified time before the adaptation step. The number behind the positive sign indicates how much later the test stimulus appeared than the adaptation step. These time intervals were adjusted by rotating the diaphragm discs against each other (Figure 1B).

As a rule, one measurement run (all specified test times) could be completed in one session (approximately 90 min). The entire measurement was performed five times for each of the nine subjects. All measurements were made at the left eye of the test persons. The experiments took place in the late morning hours.

#### 2.2.3. Evaluation of the Data

In general, the threshold stimulus strength had to be determined by interpolation, since only in exceptional cases were exactly five yes responses given at a specific test intensity. Usually, a test stimulus I_1_ with more than five yes responses was followed by I_2_ with fewer than five. The threshold strength I_s_ was then calculated according to the formula:

I_s_ = I_2_ + (I_1_ − I_2_) · (5 − n_2_)/(n_1_ − n_2_)

where: n_1_ is the number of yes responses at I_1_; n_2_ is the number of yes responses at I_2_

**Figure 2:**
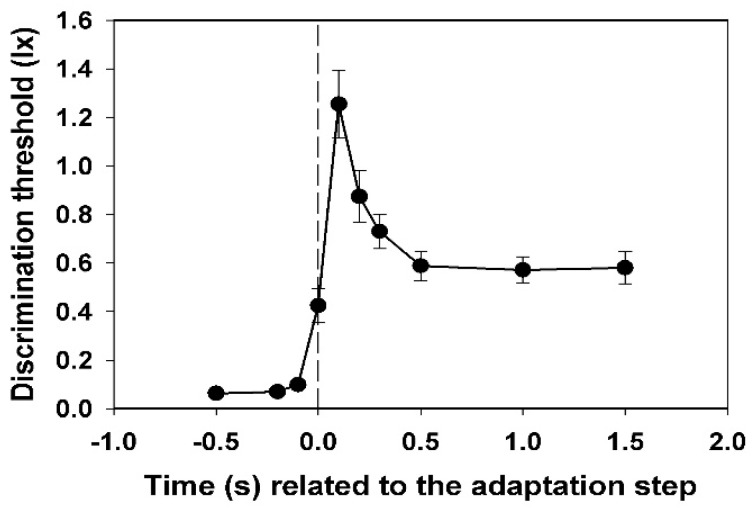
Time course of the discrimination threshold, averaged for all nine test subjects, with standard deviations. Basic adaptation light 0.5 lx, adaptation light step 2.5 lx.

The mean values and their range of confidence for each subject were calculated from the five values for the threshold intensity obtained for each test time. This approach is justified because it can be assumed that the discrimination thresholds are normally distributed [12]. The averaging of the individual time courses then gave the mean course of the discrimination threshold for all subjects (Figure 2). For this, all values obtained (always 45) were used, and the respective standard deviations were also calculated. The *t*-test was used to test for significant differences between the threshold values at the beginning of the increase.

### 2.3. Electroretinographic Studies 

#### 2.3.1. Determination of the Electroretinographic Threshold (Increment Threshold) 

##### Equipment

The system described in Section 2.1 was used to generate constant adaptation light, adaptation light pulse, and test stimuli. The ERG was recorded at the left eye of the subject with the help of a sub-lid electrode. This was a filament electrode consisting of approximately 10 metal-vaporized fibres (DTL ERG thread, UniMed Electrode Supplies, Farnham, England). The reference electrode was fixed at the left lateral corner of the eye, and the mass electrode at the left earlobe. In order to reduce the skin resistance, the skin under the reference electrode was roughened somewhat. After degreasing this site and the earlobe with alcohol, a thin layer of conductive paste was applied, and then the Ag/AgCl electrode was attached. In most cases, electrode resistances (different and indifferent electrode to ground) of less than 3 kΩ could be achieved. If the resistance was more than 5 kΩ, the procedure of applying the electrode was repeated. The electroretinograms were recorded with a two-channel amplifier and recorder for sum potentials. The amplifier had an input resistance of 200 MΩ. The measurements were carried out with a sensitivity of 25 μV/cm, an upper cut-off frequency of 20 Hz and a lower cut-off frequency of 0.1 Hz. The gain could still be changed during the evaluation of the recorded signals. The impedance of the electrodes and the input impedance of the amplifier comply with the ISCEV standards for electroretinography.

##### Subjects

A total of 12 subjects participated in the tests (four female and eight male). The age ranged between 20 and 59 years. They were, with two exceptions, persons other than those involved in recording the discrimination threshold, because of the large time interval between the two investigations. Apart from slight refractive anomalies, which were corrected during the adjustment of the device, all had normal vision. 

Ethics: All subjects gave their informed consent for inclusion before they participated in the study. The study was conducted in accordance with the Declaration of Helsinki, and the protocol was approved by the Ethics Committee of the Medical Faculty of the University of Leipzig.

##### Procedure of the Tests

After the electrodes were placed, the subject was positioned in front of the device (Figure 1A), where the basic light (0.5 lx) was set. The optical system was adjusted so that the test person saw the fixation cross sharply. Then she/he adapted to the light conditions over the course of 15 min. Only then did the measurement begin. Because of the relatively strong fluctuations of the ERG amplitudes, mean values from 10 stimulus responses were recorded. For this, the averager of the system was triggered by the test stimulus. The stimulus intensities were chosen in such a way that the b-wave amplitudes were as close as possible above and below 10 μV (threshold value), so that the threshold strength could be determined later on by interpolation. The duration of the investigation was limited to 60 min because of the limited concentration capacity of the subjects. 

Test times were: −0.5; −0.3; −0.2; −0.1; 0.0; 0.1; 0.2; 0.3; 0.4; 0.5; 1.0, and 1.5 s before and after the step of the adaptation light.

To measure an entire run from −0.5 s to + 1.5 s, 2–3 sessions were necessary.

##### Evaluation of the Data

The average courses were already being calculated during the experiment based on 10 electroretinograms. The resulting b-wave amplitudes were plotted as a function of the stimulus intensity. By linear interpolation, the stimulus intensity at which the b-wave amplitude corresponded to the threshold value of 10 μV was determined. This value represents the increment threshold at the actual time relative to the adaptation step. For the time between immediately before and after the adaptation light step, the responses to the step and test stimulus are superimposed. Here, the differences of the b-waves with and without test stimulus were calculated.

The threshold determination was carried out for all examination times and all subjects. Thereafter, the mean course for all 12 subjects with the standard deviations was calculated (Figure 4). The *t*-test was used to test for significant differences between the threshold values at the beginning of the increase.

#### 2.3.2. Measurement of the Electroretinographic Response to the 5 s Adaptation Pulse

##### Special Features of the Equipment 

In order to obtain a true response to the 5 s light pulse, a DC amplifier or an AC amplifier with a very low lower cut-off frequency is required. We used an AC EEG amplifier with a lower cut-off frequency of 0.005 Hz. This corresponds to a time constant of about 30 s. In this way, we could be sure that the ERG response to the 5 s light pulse would be recorded correctly. The remaining settings were: upper cut-off frequency 10 Hz, sensitivity 0.2 mV/V. Due to the low lower cut-off frequency and the long measuring time, the signal was superimposed by strong low-frequency fluctuations. For this reason, as many responses as possible had to be recorded and then averaged. 

##### Subjects

Since the adaptation pulse was a relatively weak stimulus (2.5 lx, 10°) and therefore only a small signal (approx. 20 μV) was to be expected, interference potentials by eye and blink movements had to be avoided as completely as possible. Because of the long duration of a single run (10 s) and the relatively large number of required runs, the investigation could only be carried out on a subject who was able to avoid blinking for a long period of time. This was subject 9.

##### Procedure of the Test and Evaluation of the Data

The preparation of the subject was carried out analogously to the description given in Section 2.3.1.1, see “Equipment”. The ERG response to the 2.5 lx, 5 s adaptation light pulse, which was repeated in the interval of 10 s, was recorded. A total of three sessions were performed, twice with 50 (test duration 8.3 min) and once with 30 (test duration 5 min) adaptation pulses. The electroretinograms were averaged during each test. The final result was obtained by a second weighted (either 50 or 30, respectively) averaging of these mean courses.

## 3. Results

### 3.1. Course of the Subjective Threshold

In Figure 2, the mean values of the subjective thresholds and their standard deviations (all subjects) are shown. Each value was calculated from 45 (nine times five) data points.

A very steep increase in the threshold is observed, which begins at −0.1 s (i.e., 0.1 s before the adaptation step). This value is already significantly different from the value measured at −0.5 s (*p* < 0.05, *t*-test). The maximum has already been reached at +0.1 s. Thereafter, a slower decay passes to the level of the new static threshold at +0.5 s.

**Figure 3:**
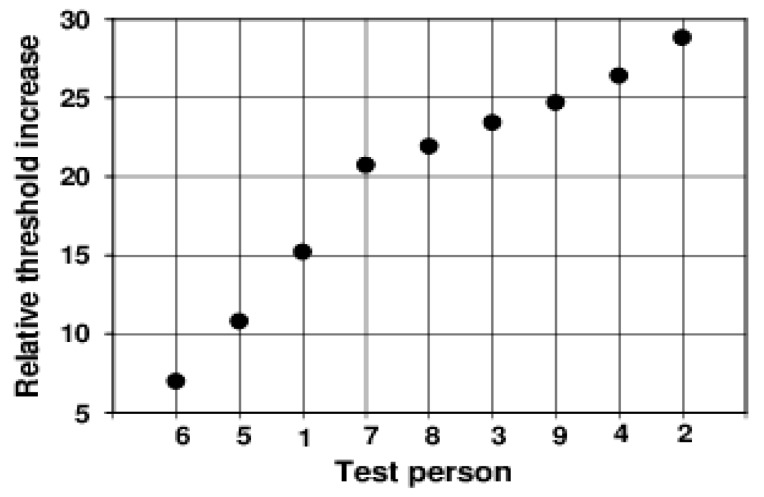
Ratio of the maximum value of the discrimination threshold to the initial threshold at −0.5 s for all subjects (ordered by the magnitude of the effect).

Individual peculiarities in the transient behaviour of the discrimination threshold hardly exist in the dynamics of the course. Only the height of the peak and the position and stability of the subsequent plateau differ from one another individually. The starting thresholds differ relatively slightly. They describe the respective subjective sensitivity at an adaptation illuminance of 0.5 lx. The maximum increase in the threshold caused by the adaptation light step of 2.5 lx, or—even better—the ratio of this value to the initial threshold at −0.5 s (relative maximum increase), could be a measure of the magnitude of the individual glare effect. These values are extremely different for the different subjects (see Figure 3). 

The relative maximum already increases over the range between 7.0 and 28.8 in this small group of test subjects. The anamnesis of our subjects provides no explanation for these large differences.

### 3.2. Course of the Electroretinographic Threshold

**Figure 4:**
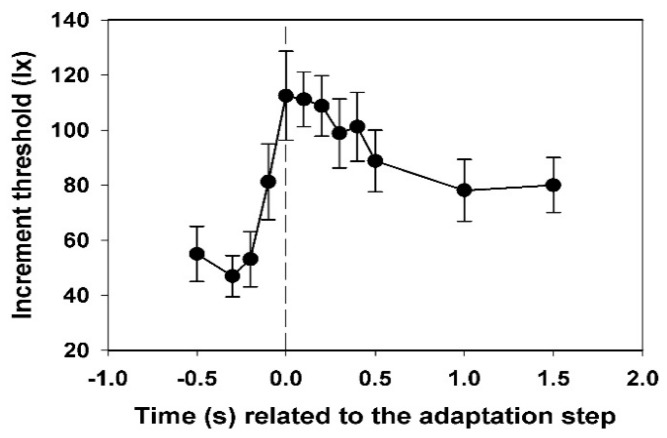
Time course of the electroretinographic increment threshold, averaged for all twelve test subjects, with standard deviations. Basic adaptation light 0.5 lx, adaptation light step 2.5 lx.

In Figure 4, the mean values and their standard deviations for the electroretinographic thresholds of all subjects are shown. Each threshold value was calculated from 12 data.

While the value for −0.2 s is not yet significantly different from that at −0.3 s, the threshold at −0.1 s is significantly increased (*p* < 0.05, *t*-test). The maximum is reached at 0.0 s, the decrease to the end-plateau is broadened in comparison to the subjective threshold. The final threshold is only reached at 1.0 s. 

### 3.3. Comparison between the Dynamics of Subjective and Electroretinographic Thresholds 

**Figure 5:**
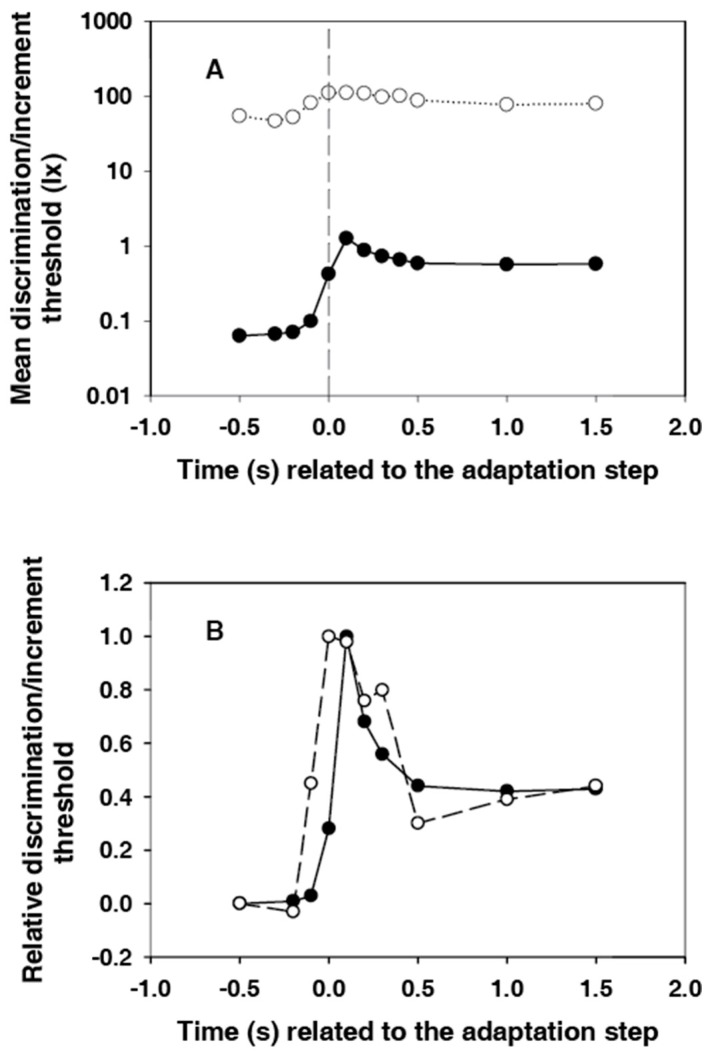
(**A**) Courses of subjective (points) and electroretinographic (circles) thresholds in the same diagram. Because of the large range to be displayed, the ordinate was graduated logarithmically; (**B**) Courses of subjective (points) and electroretinographic (circles) thresholds. They were made comparable by subtracting the initial threshold at −0.5 s and dividing by the maximum value. In both courses, the values at −0.1 s are already significantly different from the basic value of the threshold.

Figure 5A shows the courses of subjective and electroretinographic thresholds in the same diagram. The difference between the two courses is about three orders of magnitude at the beginning, after the light step it is reduced to around two orders of magnitude. Thus, the relative adaptation-induced sensitivity loss is significantly greater for the sensation than for the electroretinogram. This finding is consistent with the results of Dodt et al. [13]. They had measured the static thresholds in humans.

For a better comparison of the dynamics of the threshold courses, the values were standardized for Figure 5B. For this purpose, the initial thresholds at −0.5 s were subtracted, and the remaining differences were divided by the maximum value. The first value, which is significantly different from the initial value, is that at −0.1 s for both courses. However, the course of the subjective threshold is narrower. The maximum is in the band of the relatively wide electroretinographic maximum. The extent of the overshooting is approximately the same for both courses. 

### 3.4. The Electroretinographic Response to the 5 s Adaptation Pulse

**Figure 6:**
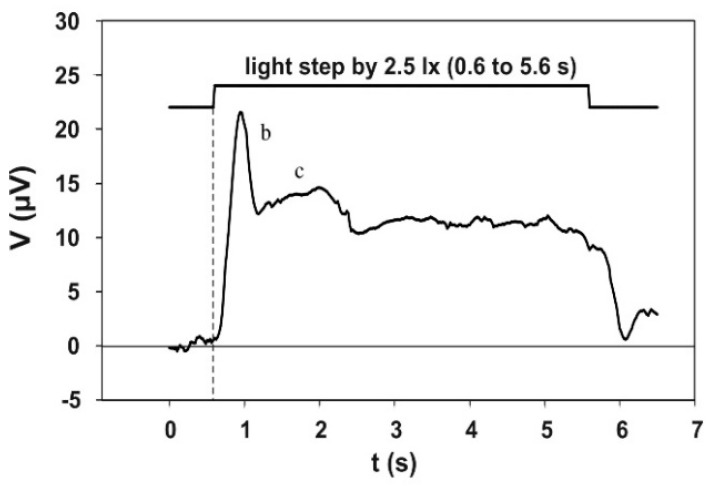
Electroretinographic response to the 5 s adaptation pulse (test subject 9, average course of 130 individual responses). The b- and the c-wave are clearly visible. The peak of the b-wave is located at about 0.3 s after the beginning of the adaptation pulse.

Figure 6 shows the electroretinographic response to the 5 s adaptation pulse. This electroretinogram was obtained by averaging 130 single runs, recorded in three sessions—twice with 50 and once with 30 runs—with the same subject. The course initially shows a pronounced b-wave, the maximum of which is reached at approximately 0.3 s after the step of the adaptation light. The b-wave is followed by a flat c-wave, which then passes into a plateau. This potential plateau ends with the end of the adaptation light pulse.

### 3.5. Comparison between the Course of the Electroretinographic Threshold and the Electroretinogram

**Figure 7:**
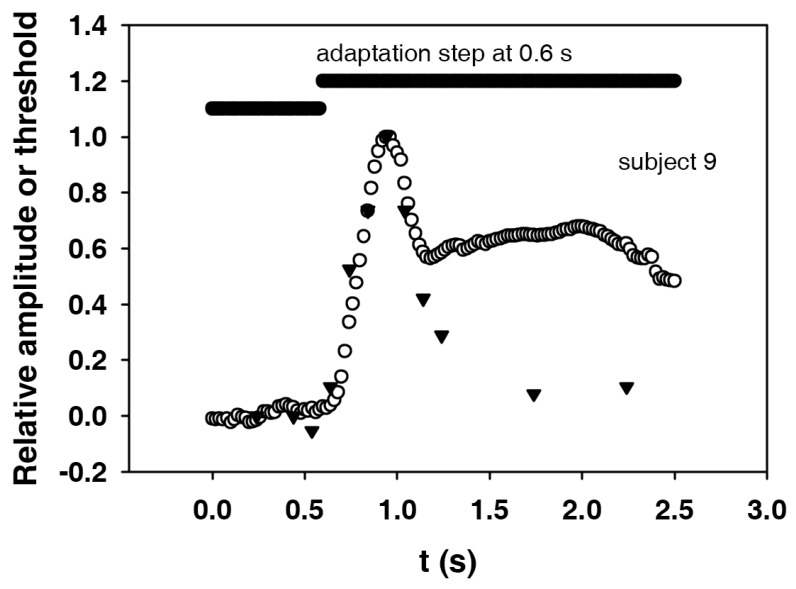
Electroretinogram (circles) and electroretinographically determined increment thresholds (triangles) for subject 9 (both courses standardized). The threshold curve has been shifted in such a way that the maximum coincides with the maximum in the electroretinogram (peak of the b-wave). This displacement to the right (to higher times) is 0.1 s.

In Figure 7, the first part of the electroretinogram (Figure 6) and the course of the electroretinographic threshold for subject 9 are shown together with the adaptation step. The maximum of the electroretinogram is at about 0.3 s, and the maximum of the threshold at 0.2 s after the adaptation step. In order to be able to compare the shapes, the course of the electroretinographic threshold was shifted by 0.1 s to the right. In addition, in order to better compare the dynamics, the initial values were subtracted and the amplitudes divided by the remaining maximum values. The result is an almost identical course for the two curves. It can not be expected that the end values (the plateaus) will also match, since the magnitude of the remaining threshold change depends on the nonlinearity of the dynamic ERG characteristics. 

## 4. Discussion

### 4.1. The Course of the Subjective Threshold (Discrimination Threshold)

In principle, our results are consistent with those of all previous investigators. Approximately 0.1 s before the adaptation step, a threshold rise starts, which quickly becomes very steep. As a rule, it reaches a maximum about 0.1 s after the step and then drops to a relatively constant value up to approximately 0.5 s after the step. This value is above the initial threshold, corresponding to the higher adaptation light. The rise in threshold decreases with increasing pre-lighting, and grows as the adaptation step increases. Authors such as [6], who recorded the thresholds over a period of more than 3 min after the adaptation step, found a re-increase of the threshold. They related this effect to the bleaching of photopigment by the adaptation step. Poot et al. [9] tried to separate the influence of the steepness of increase of the adaptation light from that of its height on the discrimination threshold. They therefore used different forms of light increase. The results show once again that receptors and neurons in the visual system are proportional and differential-quotient sensitive. The threshold curves are always in accordance with the anticipated course of the excitation. This result corresponds to our assumption.

The fact that the threshold is already significantly increased 0.1 s before the adaptation step (see Figure 2) may cause visual events that happened actually before a glare to not be noticed. As early as 1961, Lisch [14] pointed out that this effect could have relevance for the visibility of objects in road traffic. New and surprising is the finding that the degree of overshooting of the threshold is subjectively so different (Figure 3). If we assume that the magnitude of the overshooting tells us something about the glare effect, the subjectively experienced glare must be very different under the same conditions.

### 4.2. The Course of the Electroretinographic Threshold (Increment Threshold)

The measurement of the electroretinographic thresholds was much more difficult for the subjects and the examiner, and above all, more time-consuming than the determination of the subjective thresholds. For this reason, only one threshold course was obtained for each subject. Thus, the results for the single subjects are less secure than the subjective thresholds. In contrast to the subjective threshold, where the peak times were relatively constant at 0.1 s, there are strong differences for this parameter in the electroretinographic threshold. The values range from −0.1 s to 0.4 s. These large inter-individual differences lead to a broadening of the peak in the mean course (see Figure 4). As in the case of the discrimination threshold, the threshold rise begins before the step of the adaptation light. The ratio of the maximum value of the threshold to the initial value does not show as much subjective difference as the discrimination threshold (Figure 3). It ranges from 1.7 to 4.2. One can conclude from this that the large subjective differences for the glare arise at higher processing levels.

The only electroretinographic threshold course recorded earlier is shown in [2]. It was obtained on a dark-adapted subject with an adaptation light step of unknown height. Similar to our own results, a rapid threshold rise with a peak at −0.02 s was followed by a decrease to a new plateau, which was reached at 0.3 s. 

### 4.3. The Electroretinographic Response to the 5 s Adaptation Pulse

The registration of the DC components of the electroretinogram in the waking human is extremely difficult because of the large number of interfering potentials that are also picked up. Even if the subject is able to avoid blinking for a long time, the signal is superimposed by slow potentials generated in head and body. Thus, it is certainly a consequence of these difficulties that the registration of the slow potentials was carried out either in narcotized animals [15,16], narcotized humans [17], or in the isolated retina (e.g., [18,19]). Kawasaki et al. [20] have published results obtained on a waking man. However, the time constant of their amplifier was too low (0.6 s) to be able to speak of DC potentials. 

The shape of our signal is, in principle, identical with the courses recorded by the other authors. However, the special course is determined by the strength and shape of the stimulus, the position of the electrodes, and possibly also by additional subjective factors. This becomes apparent in particular in the paper of Hanitzsch et al. [17], which showed (in Figure 4) the dependence of the human DC electroretinogram on the stimulus intensity (albeit in the case of dark adaptation). The potential generated at 3.6 cd/m^2^ was very similar to the potential recorded by us. At lower stimulus levels, the initial overshoot (b-wave) decreases more and more, while at higher stimuli the plateau becomes lower and finally even negative (prevailing PIII). With regard to the c-wave, Granit [12] had, in 1933, already described very strong individual peculiarities for the cat. 

### 4.4. Comparison between the Courses of Electroretinographic Threshold and Electroretinographic Excitation 

In our assumption, it stands to reason that the dynamics of the threshold changes are determined by the course of the excitation. In order to be able to verify this for the electroretinographic increment threshold in connection with an adaptation step, the corresponding excitation, namely the electroretinographic response to the step, had to be recorded. Figure 7 shows that the courses of both events are almost identical when the peaks are synchronized. In the present case, this means that the course of the threshold had to be shifted by 0.1 s to the right, i.e., to higher times. The same dynamics of the two courses strongly suggest that our hypothesis is correct: The threshold follows the course of the excitation. From this it can be concluded that the same mechanisms that determine the course of the excitation are also responsible for the change in sensitivity. Thus, we were able to confirm a suggestion by Baker [21] and Boynton [5], according to which the rise in the threshold is due to the excitation induced by the adaptation step. Boynton [5] even assumed that the course of the threshold represents an image of the on-response on the adaptation step. 

If the threshold approximately follows the course of the excitation, it can be assumed that the course of the threshold also roughly describes the course of the excitation. Thus, the course of the discrimination threshold should also reflect the course of the visual excitation.

An attempt will now be made to explain why the threshold increase precedes the response ERG by approximately 0.1s. It can be assumed that a threshold increase occurs when the responses to the test stimulus and to the adaptation step begin to overlap. From then on, due to the nonlinearity of the characteristic curve, the increase in b-wave amplitude generated by the test stimulus becomes smaller; or, rather, the test stimulus amplitude must be increased in order to achieve the same gain of the b-wave. If we assume approximately the same latencies for the responses to the adaptation step and the test stimulus, the overlay begins as soon as the time between test stimulus and adaptation step becomes smaller than the duration of the response to the test stimulus, which is the duration of the b-wave. The width of the b-wave triggered by our test stimuli was actually about 0.1–0.2 s. This explains why the electroretinographic threshold already rises before the adaptation step. For the same effect at the visual threshold, Crawford gave two possible explanations. One is that the excitation triggered by the strong step in adaptation light overtakes that by the weak test stimulus on the way to the brain. His second explanation agrees with our results obtained at the ERG: Since both excitations have a certain duration, they overlap. This leads to the threshold increase. Generalizing, one can say: The early rise in threshold occurs whenever two excitations of a certain duration are superimposed in a system with a nonlinear characteristic. This applies to all stations of the visual system from the retina to the visual cortex.
